# Application of modified-alginate encapsulated carbonate producing bacteria in concrete: a promising strategy for crack self-healing

**DOI:** 10.3389/fmicb.2015.01088

**Published:** 2015-10-13

**Authors:** Jianyun Wang, Arn Mignon, Didier Snoeck, Virginie Wiktor, Sandra Van Vliergerghe, Nico Boon, Nele De Belie

**Affiliations:** ^1^Magnel Laboratory for Concrete Research, Department of Structural Engineering, Ghent UniversityGhent, Belgium; ^2^Laboratory of Microbial Ecology and Technology, Department of Biochemical and Microbial Technology, Ghent UniversityGhent, Belgium; ^3^Polymer Chemistry and Biomaterials Group, Department of Organic Chemistry, Ghent UniversityGhent, Belgium; ^4^Microlab, Faculty of Civil Engineering and Geosciences, Delft University of TechnologyDelft, Netherlands

**Keywords:** modified-alginate hydrogel, *B. sphaericus* spores, bacterial CaCO_3_, *in situ* activity, oxygen consumption, crack self-healing

## Abstract

Self-healing concrete holds promising benefits to reduce the cost for concrete maintenance and repair as cracks are autonomously repaired without any human intervention. In this study, the application of a carbonate precipitating bacterium *Bacillus sphaericus* was explored. Regarding the harsh condition in concrete, *B. sphaericus* spores were first encapsulated into a modified-alginate based hydrogel (AM-H) which was proven to have a good compatibility with the bacteria and concrete regarding the influence on bacterial viability and concrete strength. Experimental results show that the spores were still viable after encapsulation. Encapsulated spores can precipitate a large amount of CaCO_3_ in/on the hydrogel matrix (around 70% by weight). Encapsulated *B. sphaericus* spores were added into mortar specimens and bacterial *in situ* activity was demonstrated by the oxygen consumption on the mimicked crack surface. While specimens with free spores added showed no oxygen consumption. This indicates the efficient protection of the hydrogel for spores in concrete. To conclude, the AM-H encapsulated carbonate precipitating bacteria have great potential to be used for crack self-healing in concrete applications.

## Introduction

Bacteria are able to mediate the precipitation of minerals, either by biologically controlled mineralization like the formation of magnetite by magnetotactic bacteria (Frankel et al., 1998; Frankel and Bazylinski, 2003), or by biologically induced mineralization which are the dominant processes among bacteria. Under suitable conditions, most bacteria are able to induce the precipitation of carbonate (Boquet et al., 1973). This biogenic precipitate is environmentally friendly, durable and compatible with building materials; due to this, microbial induced carbonate precipitation (MICP) is being widely investigated for civil engineering applications, such as surface protection of building materials (Rodriguez-Navarro et al., 2003; Tiano et al., 2006; Jimenez-Lopez et al., 2007; De Muynck et al., 2008), sand cementation (DeJong et al., 2006; Van Paassen et al., 2010; Cheng et al., 2013), crack repair (Ramachandran et al., 2001; Jonkers et al., 2010; Van Tittelboom et al., 2010; Wiktor and Jonkers, 2012), etc.

Concrete is the most widely used construction material. However, due to its inherent heterogeneity, low tensile strength and non-ideal service environment, concrete is quite susceptible to cracking. Cracks in concrete are the main cause for a reduced durability as they provide an easy path for water and aggressive substances to penetrate into the concrete matrix. Therefore, it is of crucial importance to do crack repair. Nowadays, a promising repair method by self-healing process is being strived for. Repair/healing agents are pre-added into concrete during the process of mixing. When cracking occurs, healing agents will be released to heal the cracks. Compared with conventional manual repair, self-repair has a distinctive advantage to greatly reduce the high maintenance and repair cost (Van Breugel, 2007). Various materials are being investigated for the self-healing aim (Dry, 2000; Kishi et al., 2007; Van Tittelboom et al., 2011; Snoeck et al., 2014a), among which MICP has attracted high attention due to its environmental friendliness, long-term viability and good compatibility with the concrete matrix.

The principle of microbial-based self-healing concrete is that carbonate precipitating bacteria together with nutrients and precipitation precursors are added into concrete during the mixing process. Upon cracking, bacteria in/around the crack surface will be activated and will precipitate CaCO_3_ to *in situ* heal the crack. Challenges rise when applying the micro-scale bacteria into the macro-scale concrete: the pH in concrete is as high as 12.5–13, which is a harsh condition for bacteria. Moreover, the cement-based matrix gradually becomes a dense structure due to ongoing hydration. Most of the pore sizes are smaller than 0.5 μm, while the size of bacteria is in the range of 1∼3 μm. Therefore, there is a big chance that bacteria inside the matrix would be squeezed and crushed. Regarding these conditions, alkaliphilic/alkali-tolerant spore forming strains should be used since spores are much more resistant and have much longer survival time than vegetative cells (Setlow, 1994, 2000). In this study, *Bacillus sphaericus*, an ureolytic, alkali-tolerant spore-forming strain was used. This strain is able to precipitate CaCO_3_ in its micro-environment by decomposition of urea [CO(NH_2_)_2_] into ammonium (NH_4_^+^) and carbonate (CO_3_^2-^). The latter subsequently promotes the bacterial deposition of CaCO_3_ in a calcium rich environment (Dick et al., 2006). In order to protect bacteria from the harsh condition, encapsulation of bacteria prior to the addition is preferable. A suitable carrier should have a ‘shell’ function, no hindering effect on bacterial carbonate precipitation and no/limited negative effect on concrete matrix.

In our earlier research, bacteria-based self-healing systems have been developed by the use of glass capillaries, porous powders, microcapsules and hydrogel encapsulated bacteria (Wang et al., 2012a,b, 2014a,b,c). Hydrogels are found to be the most suitable for general practical occasions. Water is an essential element for bacterial activity. Therefore, in order to obtain crack healing in a bacteria-based healing system, sufficient and continuous water supply to activate and to keep bacterial activity in the crack zone is an important determining factor. In most of the above mentioned systems, this was achieved by full-time immersion or part-time immersion (16 h in water and 8 h exposed to air, see Wang et al., 2014a), except for the one using hydrogel as bacterial carrier which was accomplished by wet-dry cycles with a limited wet period (1 h wet and 11 h dry, much closer to the realistic conditions, see Wang et al., 2014c). This is due to the hydrogel having a high water absorption and retention capacity. The water absorbed in the wet stage can retain inside the hydrogel matrix and can be gradually released and support bacterial activities (germination, bio-precipitation) during the dry stage. Therefore, the water supply frequency can be greatly reduced. In previous work, a pluronic-based hydrogel was applied and healing superiority was obtained (Wang et al., 2014c). However, the main drawback of this type of hydrogel is that the compressive strength of the specimen was drastically decreased by about 50%, which is not acceptable in practice.

In this study, a new type of alginate-based hydrogel was investigated for its potential use as bacterial carrier in the bacteria-based self-healing system. Sodium alginate is a water-soluble anionic polysaccharide extracted from the cell walls of brown algae. It is a linear copolymer composed of mannuronic and guluronic acid, covalently linked in varying sequences and blocks and is commercially available as a sodium salt (NaAlg). When NaAlg is combined with multivalent cations such as calcium (originating from salts such as calcium chloride, CaCl_2_), a physically cross-linked network is formed, which becomes insoluble in water. Calcium alginate is widely used for encapsulation of bacteria and enzymes due to its good biocompatibility (Viveganandan and Jauhri, 2000; Blandino et al., 2003; Dey and Roy, 2011; Dong et al., 2014). However, the bonds between the calcium cations and the carboxylate groups in alginate are ionic, formed by electrostatic interaction. Covalent bonds, on the other hand, consist of two atoms sharing a pair of electrons. This bond is much stronger and requires relatively high energies to break. As such, for application in concrete, in this study, instead of the calcium alginate, the sodium alginate will be modified chemically to incorporate double bonds and with UV polymerization to create the possibility of forming a covalently bonded modified alginate (AM). This will be a stronger carrier for bacteria compared to calcium alginate.

Firstly, viability and precipitation tests were performed to verify whether bacteria spores were still viable and can precipitate CaCO_3_ after being encapsulated into the AM hydrogel. Meanwhile, the swelling capacity and the influence of hydrogel on concrete strength were examined. Subsequently, the hydrogel encapsulated bacterial spores were incorporated into mortar specimens and *in situ* bacterial activity on damaged specimens was monitored.

## Materials and Methods

### Bacterial Strain

*Bacillus sphaericus* LMG 22257 (Belgian Coordinated Collection of Microorganisms, Ghent) was used based on previous research (Dick et al., 2006). This strain has a high urease activity (40 mM urea hydrolyzed. OD^-1^ h^-1^, OD: optical density) and can produce CaCO_3_ in a simple and controllable way (Wang, 2013). *B. sphaericus* spores were cultivated in the liquid minimal basal salts (MBSs) medium (Kalfon et al., 1984). Mature spores were transferred as the inoculum (1% v/v) into the sterile MBS medium. The culture was incubated at 28°C on a shaker (100 rpm) for 14–28 days until more than 90% of the cells were spores. The spores were then harvested by means of centrifugation (7000 rpm, 4°C, 7 min, Eppendorf MiniSpin, Hamburg, Germany). The supernatant was removed and the paste of spores was re-suspended in a physiological solution (NaCl, 8.5 g/L). The spores suspension was then subjected to pasteurization to minimize the amount of vegetative cells. Afterward, it was stored in a 4°C fridge for experimental use. The concentration of the spores in the suspension was about 2.3 × 10^9^ spores/mL, determined by plate counting.

### Synthesis of Methacrylate Modified Alginate

The synthesis of methacrylate modified alginate (AM) was adapted from literature (Smeds and Grinstaff, 2001; Rouillard et al., 2011). The chemicals needed were sodium alginate, methacrylic anhydride (MAA) and sodium hydroxide (NaOH), which were purchased from Aldrich (Bornem, Belgium) and were used as received. Dialysis membranes Spectra/Por^®^4 (MWCO 12,000–14,000 Da) were obtained from Polylab (Antwerp, Belgium). The procedure of synthesis was as follows. Sodium alginate powders (20 g) were first dissolved in 1 L double-distilled water. After that, MAA (two equivalents with respect to the alcohol functionalities present) was added. To keep the pH at 8, a 5 M NaOH solution was added. The reaction mixture was stirred vigorously at room temperature overnight. Dialysis was performed for 3 days after diluting the mixture with 1 L double-distilled water in order to remove the unreacted agents. Finally, the purified product modified alginate (AM) was freeze-dried (Christ Alpha 2–4 LSC Freeze Dryer) for further experimental use.

### Encapsulation of *B. sphaericus* Spores into AM Hydrogel

#### Synthesis of AM Hydrogel

The polymer chain of AM needs to be crosslinked into a hydrogel network to entrap the bacterial spores. This was realized by applying UV irradiation in the presence of an initiator (Irgacure^®^2959) during the synthesis. The initiator solution (5 g/L, 5 mL) was added into AM solution (0.5 g AM with 10 mL water) after the AM was completely dissolved. The whole mixture was kept mixing for another 10 min and was subsequently degassed for 5 min. Care should be taken to avoid exposing the UV initiator to light. Therefore, the mixture was wrapped in aluminum foil. Afterward, the mixture was injected into a chamber made of two glass plates separated by a 1 mm thick silicone spacer. The glass plates were then clamped and subjected to UV irradiation for 1 h. After UV irradiation, the solution in the chamber formed a gel-like sheet. And a reference AM hydrogel was obtained.

#### Encapsulation of *B. sphaericus* Spores into AM Hydrogel

In the same way, the initiator solution (5 g/L, 5 mL) was added into an AM solution (0.5 g AM with 9 mL water) after the AM was completely dissolved. The solution was kept mixing for another 10 min. Afterward, 1 mL *B. sphaericus* spores suspension was added and the whole mixture was stirred for 10 min. Next, it was degassed for 5 min. The subsequent steps were the same as the procedure for production of the reference AM hydrogel sheet. In this study, the AM hydrogels with and without encapsulated bacterial spores were represented as AM-HS and AM-H, respectively.

Both AM-H and AM-HS sheets were subjected to freeze drying and then grinded to obtain dry powders (20–100 μm) for experimental use. Theoretically, there were around 4.4^∗^10^9^ spores present in 1 g AM-HS powders.

### Viability of *B. sphaericus* Spores after Encapsulation

#### Urease Activity of Spores after Encapsulation

In order to investigate whether the spores can germinate and revive urease activity after encapsulation, 0.1 g AM-HS powders were added into 100 mL sterile medium (YU medium) consisting of yeast extract (YE; 20 g/L) and urea (20 g/L). The amount of urea decomposed was used to evaluate the viability of the encapsulated spores, which was calculated based on the total ammonium nitrogen (TAN) measured in YU medium. One mole of urea [CO(NH_2_)_2_] produces two mole NH_4_^+^. The amount of NH_4_^+^ can therefore indicate the amount of urea decomposed. TAN concentration was measured colorimetrically by Nessler method (Ivanov et al., 2005). As controls, 0.1 g AM-H powders and an equivalent (equivalent to the amount of the spores in 0.1 g AM-HS powders) amount of free spores (0.2 mL spores suspension) were also added into the same medium. The amount of urea decomposed in YU medium after 1 and 3 days was measured. The experiments were performed in triplicate (*n* = 3).

#### Calcium Carbonate Precipitation by Encapsulated Spores

Small AM-HS pieces (0.1 g, without grinding) were added to 100 mL precipitation medium, which consisted of urea (20 g/L), Ca(NO_3_)_2_.4H_2_O (11.8 g/L) and YE (20 g/L). The pH of the medium was adjusted to 7 by use of NaOH (1 M). After 3 days, the amount of urea decomposed in the medium was measured by the above mentioned TAN method. Meanwhile, the hydrogel pieces were taken out from the medium and were dried at room temperature for another 3 days. Thermogravimetric analysis (TGA) was used to further demonstrate the formation of CaCO_3_ in/on the hydrogels. Dry hydrogel pieces were put in the TGA instrument (TA instruments TGA Q50). The temperature was increased from room temperature to 900°C at a speed of 10°C/min in nitrogen atmosphere. The weight loss of the sample during heating was recorded and was shown in a weight-temperature graph. AM-H was also examined as a control. Meanwhile, the morphology of the precipitation was studied by use of a Phenom FEI scanning electron microscope (SEM). The samples were first gold coated (Emitech K550X) before SEM examination. The experiments were performed in triplicate (*n* = 3).

### Leakage of the Spores from AM-HS

The leakage test was performed to evaluate the cell entrapping property of the hydrogel. Hydrogel powders (0.15 g, AM-H or AM-HS) were added to a falcon tube with 20 mL sterile saline (8.5 g/L NaCl) inside. The falcon tube was first subjected to the vortex for 2 min (to mimic the mixing process during mortar casting) and was then kept static at the temperature of 20°C. After 3 min, the hydrogel powders settled down. The 1 mL suspension from the falcon tube was added to 9 mL sterile saline in a test tube, which was then subjected to the vortex for 5 s. Subsequently, 1 mL suspension was taken from the first test tube and transferred to the second test tube and vortexed. The same operation was repeated three times (five dilutions in total, decimal dilution series). After that, 100 μL suspension was taken from each test tube (from the dilution series of 10^-5^ to 10^-1^) and was spread on an agar plate (pH = 9) homogeneously by a Drigalski spatula. All the agar plates were then put upside down in the 28°C incubator. The colonies on the agar plates were counted after 48 h and the amount of spores leached out could be calculated. Samples were also taken from the falcon tube to examine the leakage after 1 and 3 days. Before sampling, the falcon tube was gently shaken for 5 s and 1 mL suspension was taken for quantifying the amount of the spores leaked. The subsequent procedures were the same as mentioned above. One replicate was used in the experiment (*n* = 1).

### Swelling Properties of AM-H

The swelling characteristics were determined by using the filtration method as described in Snoeck et al. (2014a). A certain mass (0.2–0.5 g) of AM-H powders was weighed and 100 ml of fluid was added. Two fluids were tested: de-mineralized water and cement filtrate. The latter was obtained by mixing 10 g of CEM I 52.5N with 100 g of de-mineralized water for 24 h and subsequent filtration. The ion-rich filtrate had a pH of 12∼12.5 and was used to mimic the condition of concrete. After fluid addition, the AM-H powders were able to take up the fluid. After 24 h, the whole mixture was filtered and the water passing the filtration was subtracted from the added amount. This result was divided by the used amount of AM-H to express the absorption capacity of the hydrogel. The tests were repeated in fourfold (*n* = 4).

Additionally, the swelling and re-swelling behavior of the hydrogel was studied. Swollen AM-H particles to full extent were vacuum dried for 1 week until constant mass. The filtration test was then conducted once more and the absorption capacity was determined. The re-swollen AM-H particles were then again vacuum dried and the second re-swelling capacity was calculated. This test was done in threefold (*n* = 3).

### Dynamic Vapor Sorption Test

Dynamic vapor sorption (DVS) measurements were applied to determine the moisture uptake capacity of the hydrogel at different relative humidities (RHs). The absorbed moisture can also be used for bacterial activity and hence, a strong moisture uptake capacity indicates that the bacteria-based healing system will also work in the occasion without extra water supply. The equipment consists of a Cahn microbalance, a temperature-controlled housing and mass flow controllers which control the appropriate flow of the wet and dry N_2_ gas. The benefit of this technique is the control over both the RH as well as the temperature (i.e., 21°C). For the DVS analysis, ∼5–10 mg of freeze-dried hydrogel was placed in the sample pan. A first step (RH of 0%) was necessary to start with a completely dry material. Afterward, the humidity was varied in systematic steps (i.e., 20, 40, 60, 80, 90, and 98%). Every subsequent step was initiated when the change of the sample mass as a function of time was lower than 0.02 mg/min. After an equilibrium value was obtained at the highest RH (98%), desorption was realized in consecutive RH steps similar to the sorption process until full desorption.

### Mortar Prisms with Hydrogels

#### Influence of AM-H on the Strength of Mortar

Series of mortar specimens were made to investigate the effect of AM-H on the mechanical properties of mortar. The reference mortar mixture was composed of Portland cement (CEM I 52.5 N), standardized sand (DIN EN 196-1 Norm Sand) and tap water. The sand-to-cement ratio was 3 and water-to-cement ratio (w/c) was 0.5. In another two mixtures, 0.5 and 1% AM-H powders versus cement weight were added, which was expressed as 0.5 and 1 m%. All compositions were mixed according to the standard NBN EN 196-1 (2005).

A flow test was performed to investigate the influence of AM-H on the workability of the mortar mixture. According to the standard En 1015-3 (1999), the fresh mortar was filled in a mold (60 mm in height, internal diameter: base 100 mm – top 70 mm), which was placed in the center of the flow table. Then the mold was removed and let the mixture elapse on the table. After 15 s, the table was jolted 15 times at a rate of one jolt per second. The diameter of the spread mortar was measured in two perpendicular directions. The average value of the two measurements was the flow value of the fresh mortar.

After the flow test, the mortar specimens were casted and stored at a RH of more than 90% and a temperature of 20 ± 2°C until the age of testing. The mechanical properties were studied at 28 days after casting. The flexural strength was measured by means of a three-point-bending test on three 40 mm × 40 mm × 160 mm mortar beams and the compressive strength was measured by means of a compression test on the obtained halves (contact area 40 mm × 40 mm), by using the testing machine Walter+Bai DB 250/15 (NBN EN 196-1, 2005).

#### *In Situ* Bacterial Activity in Mortar

AM-H encapsulated spores were added into mortar specimens (40 mm × 10 mm × 160 mm) to investigate their *in situ* activity upon cracking. As a control, un-encapsulated spores and AM-H were also incorporated into the specimens. The composition of the prisms of each series is shown in **Table 1**. All mortar prisms were made by using the same cement, sand, and water as mentioned above. Urea and YE were added as bacterial nutrients. Group N are the prisms with only bacterial nutrients added. Group N+AM-H are the ones with bacterial nutrients and hydrogels without spores loaded (AM-H). Groups N+AM-HS and N+S are the specimens with bacterial nutrients, and encapsulated spores or free spores, respectively. The addition of hydrogel powders was 2% of the cement by weight. The amount of free spores incorporated into the prisms was equivalent to the amount of the spores in AM-HS powers (added to the specimens N+AM-HS). Urea and YE were dissolved in the water before being added to the mixture. Hydrogel powders (AM-H or AM-HS) were first mixed with cement for 15 s at the speed of 140 rpm and then 5 s at 285 rpm. After that, water which contained dissolved with urea and YE, was added into the mixer. Free spores were also first mixed with water and then were added to the mixer. Subsequent steps were performed according to the standard NBN EN 196-1 (2005). Two prisms were made for each series (*n* = 2). After casting, the molds were placed in a moisture room (20°C, >90% RH). The specimens were demolded after 24 h and were then stored in the same moisture room until the testing age.

**Table 1:** Composition of the mortar prisms in each series.

Type	Cement (g)	Sand (g)	Water (g)	Urea (g)	YE (g)	AM-H (g)	AM-HS (g)	Spores suspension (mL)
*N*	80	240	40	3.2	0.68	0	0	0
N+AM-H	80	240	40	3.2	0.68	1.6	0	0
N+AM-HS	80	240	40	3.2	0.68	0	1.6	0
N+S	80	240	36.8	3.2	0.68	0	0	3.2

**FIGURE 1:**
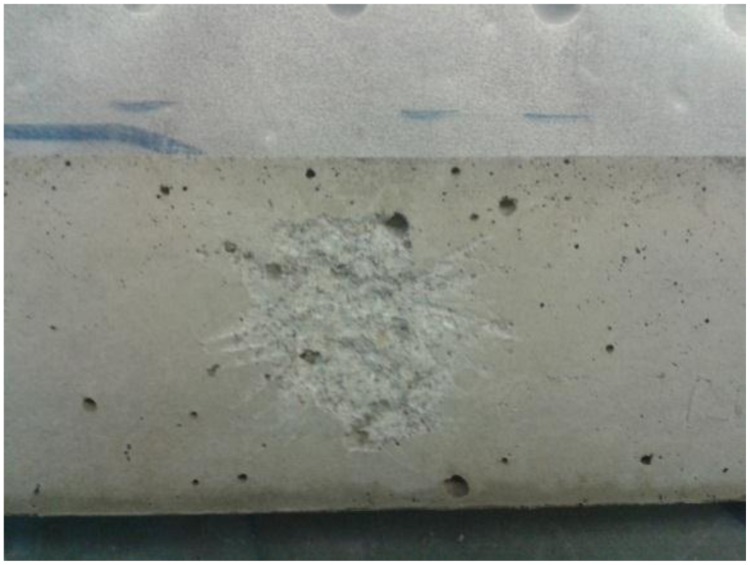
**Scratched surface of the prism**.

**FIGURE 2:**
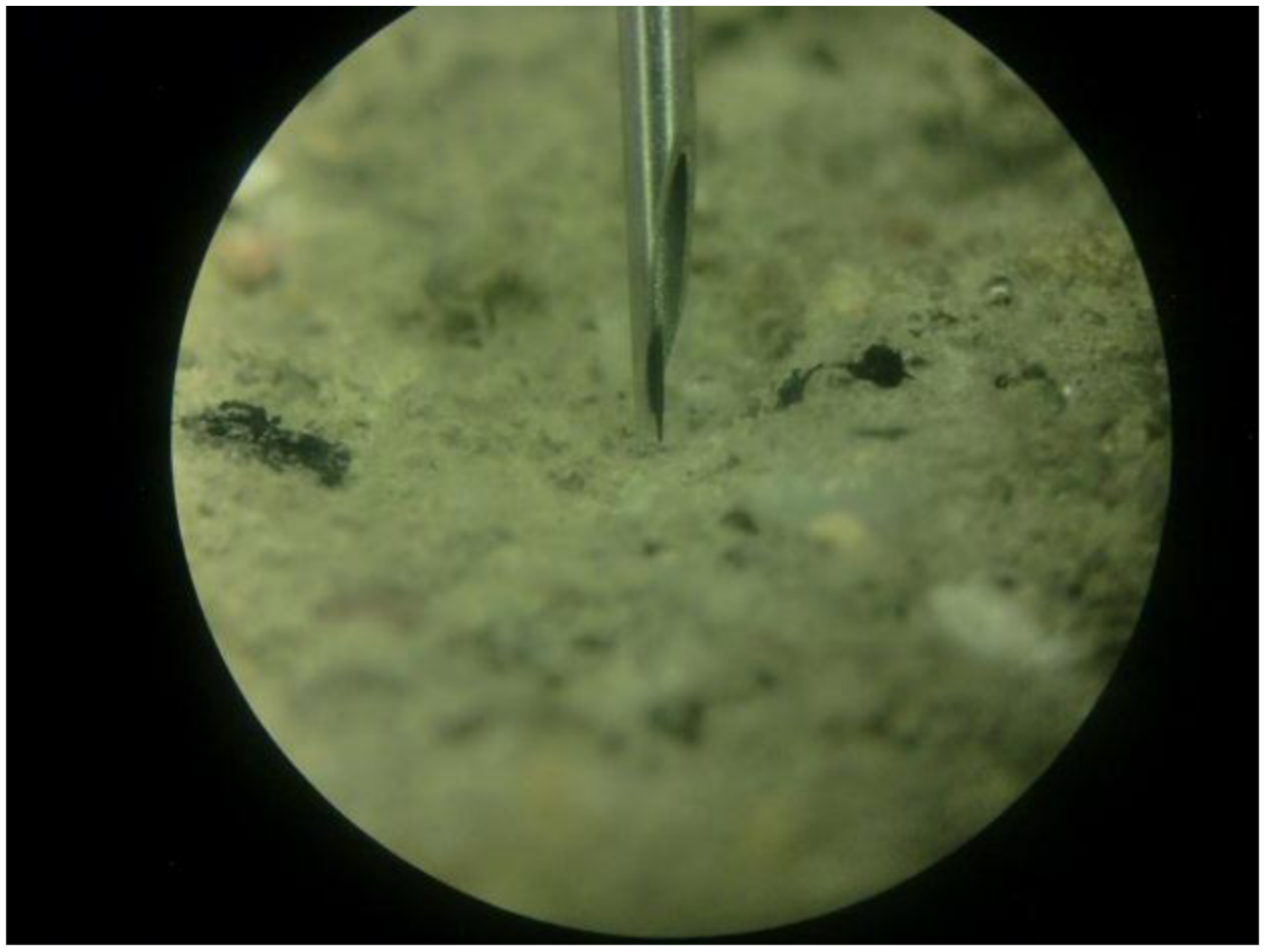
**Sensor tip just above the surface of the prism**.

Oxygen is consumed during the germination of *B. sphaericus* spores. Therefore, whether the spores (encapsulated or not encapsulated) in/on the crack surface become active or not can be indicated by the oxygen consumption on the crack surface. An optical oxygen microsensor (NTH-PST1-L5-TS-NS40/0.8-YOP, needle type housing microsensor with a tip diameter < 50 μm, Presens, Germany) was used in this study to measure the oxygen concentration. After 28 days, the prisms were taken out from the moisture room and subjected to the oxygen consumption test. In order to mimic a crack surface (damaged surface), part of the surface of each prism was slightly scaled off (shown in **Figure 1**). The prism was then immediately fully immersed into water with the damaged surface on the top. The microsensor was fixed to a motorized micromanipulator on the vertical axis. Automatic oxygen consumption profiles were measured in vertical steps of 50 μm (0.1 μm resolution), from 5 mm above the damaged surface, toward the surface. For the prisms with hydrogels incorporated, the sensor was just above a hydrogel particle and its tip should be as close as possible to the surface on the last step (**Figure 2**). More details regarding the oxygen measurement procedure can be found in the references (Mock et al., 2002; Presens, 2006; Wiktor and Jonkers, 2011). It should be noted that the sensor tip was always under water during the whole measuring process.

## Results

### Viability of AM-HS

#### Germination and Ureolytic Activity of the Spores after being Encapsulated into the Hydrogel

**FIGURE 3:**
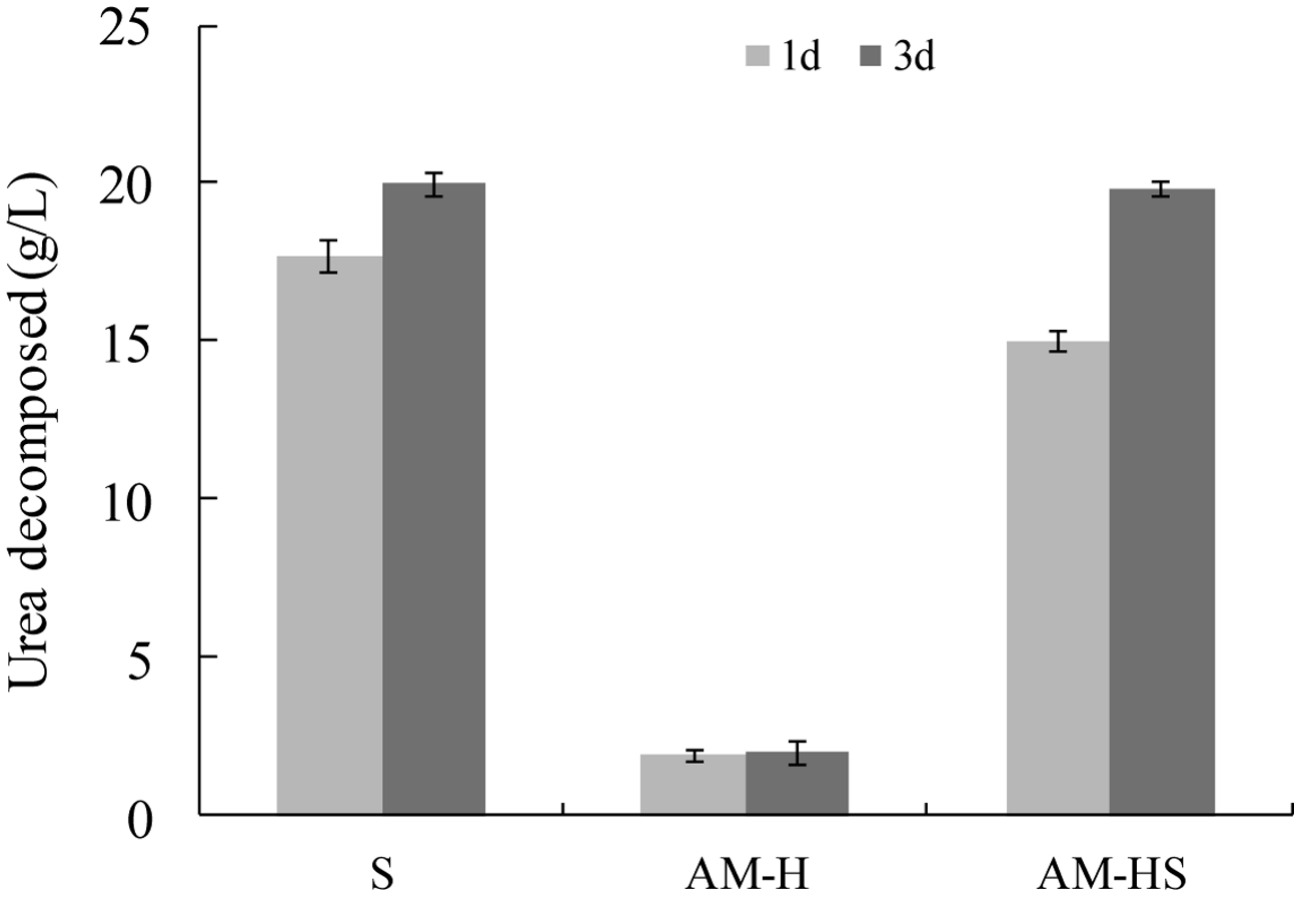
**Ureolytic activity of non-encapsulated spores (S), modified alginate hydrogel (AM-H), and modified alginate hydrogel encapsulated spores (AM-HS; *n* = 3)**.

Spores were still viable after being encapsulated into AM-H. As shown in **Figure 3**, around 15 g/L urea was decomposed by the encapsulated spores in the first day. Non-encapsulated spores had a higher ureolytic activity in the first 24 h (about 18 g/L urea was decomposed). However, the urea in both media (20 g/L), either containing free spores or encapsulated spores, was completely decomposed after 3 days. The media with AM-H added also had small amount (about 2 g/L) of urea decomposed. This is due to the sterilization process: by autoclaving the YU medium at 120°C for 20 min, a small amount of urea was decomposed during the heating process. Besides, the hydrogels were non-sterile, which might also cause some contamination, resulting in a small amount of urea decomposition. Nonetheless, the amount of urea decomposed in the medium with AM-H was quite limited compared to that with AM-HS.

#### Calcium Carbonate Precipitation by AM-HS

After 3 days, the urea decomposed in the precipitation medium with AM-H and AM-HS added was 0.6 ± 0.3 and 17.9 ± 0.5 g/L, respectively. Encapsulated spores decomposed about 90% of the urea in the medium. In addition, many white–yellowish precipitates were found on the hydrogel slices and in the bulk solution with AM-HS, while almost no precipitates were found in the medium with AM-H. The very low amount of urea decomposition in the medium with AM-H could be due to the contamination from non-sterile hydrogels.

**FIGURE 4:**
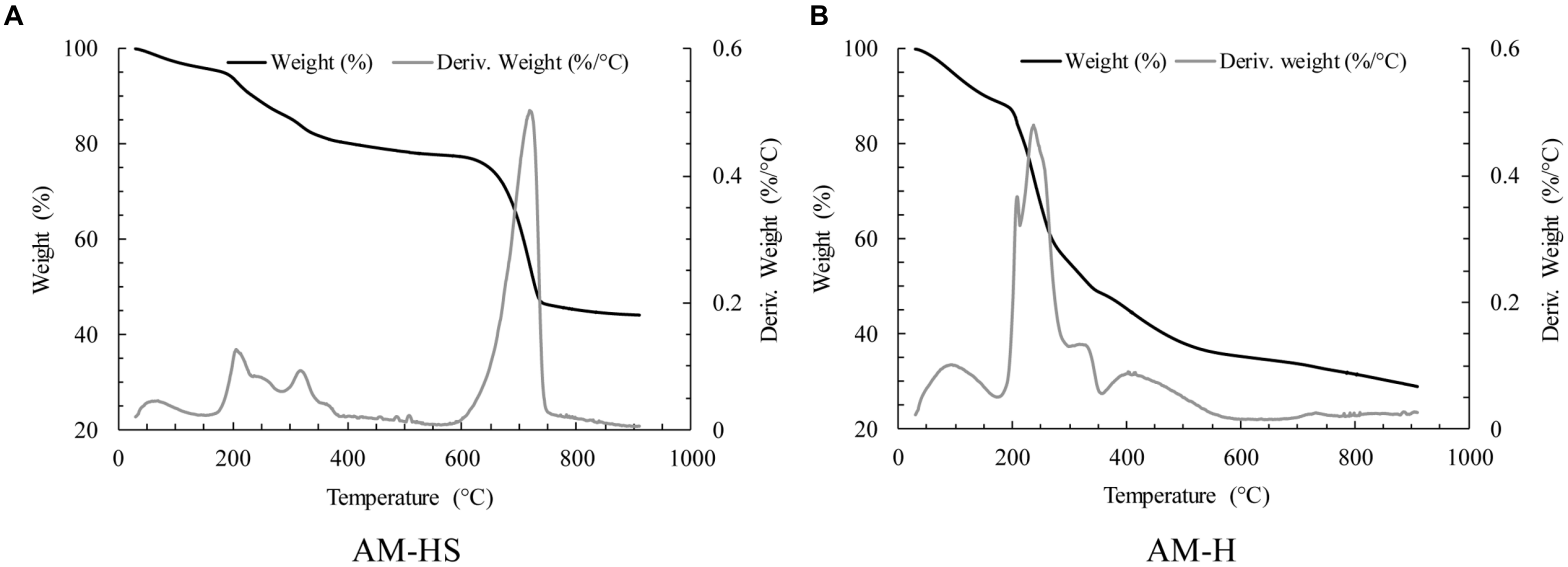
**Thermogravimetric analysis (TGA) graphs of the samples from AM-HS **(A)** and AM-H **(B)** pieces after being immersed in the precipitation medium**.

The precipitates on the hydrogels were proven to be calcium carbonate (CaCO_3_) by TGA analysis. As shown in **Figure 4A**, the weight of the sample continuously decreased from room temperature till about 800°C. The weight loss before 200°C was caused by the dehydration of the samples, while weight decrease between 200∼400°C was due to the degradation of the hydrogel. A sharp weight loss (about 30% of the initial sample weight) occurred in the temperature range of 650∼800°C. This was attributed to the CO_2_ emission from the decomposition of CaCO_3_ (Oniyama and Wahlbeck, 1995). The average percentage of CaCO_3_ precipitation on the hydrogels was around 70% by weight (based on the results from three samples). Similarly, due to the dehydration and the degradation of the hydrogel, weight decrease before 200°C and between 200∼400°C can also be seen in the graphs of the sample AM-H (**Figure 4B**). Obviously, the weight loss percentage from hydrogel degradation in AM-HS was much lower than that in AM-H due to the presence of CaCO_3_ precipitation. It was noticed from the derivation curve of **Figure 4B** that a very slight weight loss occurred between 700∼750°C. This weight loss (around 2%) could be due to the decomposition of carbonate, which is the intermediate product from the heating process of the alginate (Soares et al., 2004; Kong et al., 2009).

**FIGURE 5:**
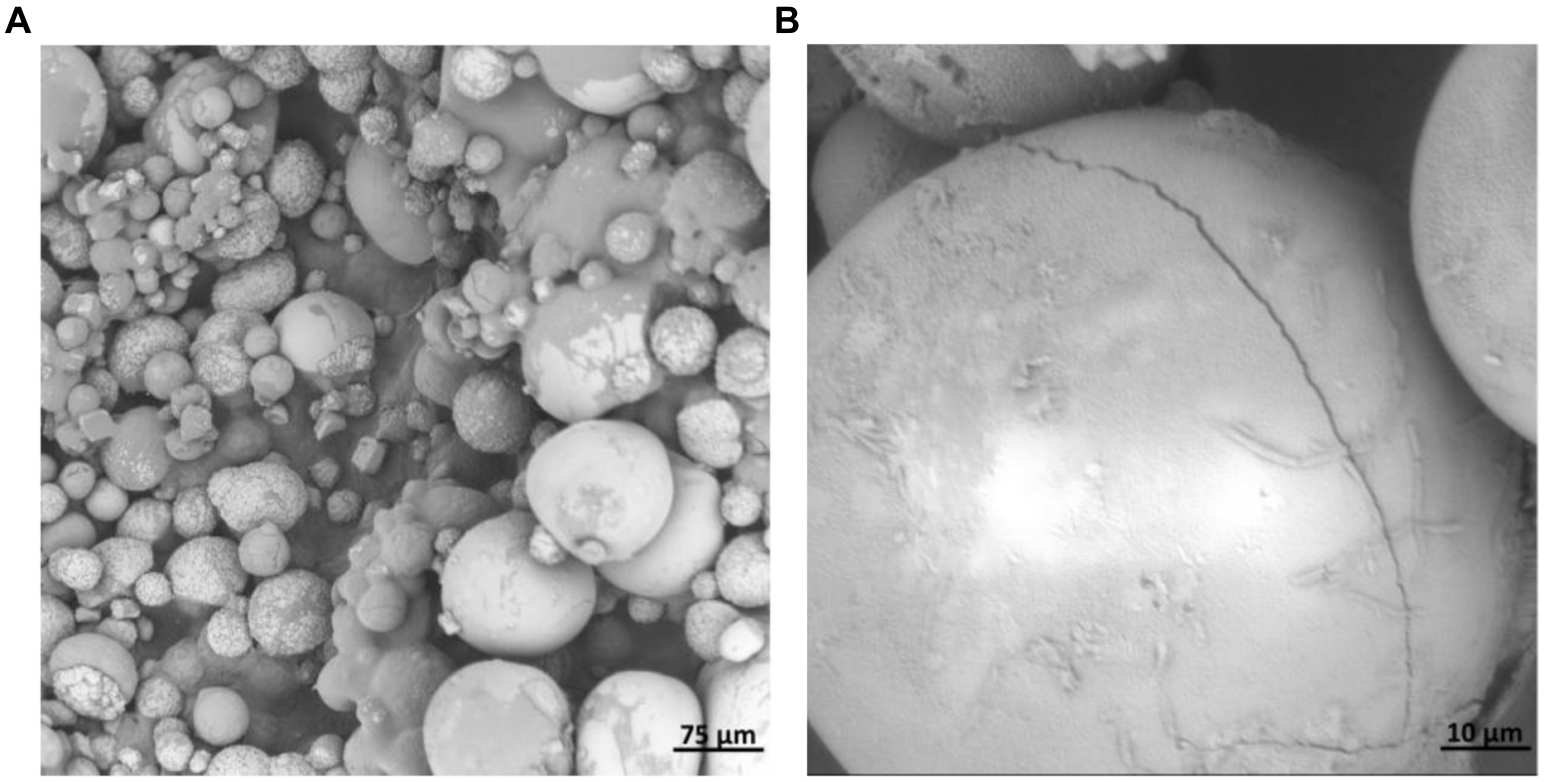
**Scanning electron microscope (SEM) images of the CaCO_3_ precipitation on/in alginate hydrogel matrix under different magnification [(A) 395x; (B) 2860x)**.

The morphology of the CaCO_3_ precipitation is shown in **Figure 5**. It can be seen that the precipitation was formed all over the surface of the hydrogel. Some of the crystals were embedded inside the matrix. The particles were mostly spherical, with smooth or rough surfaces. The size was quite heterogeneous, varying from 5 to 80 μm. In **Figure 5B**, bacterial indents (about 2∼3 μm long) on the surface of some particles can be clearly seen.

Calcium carbonate has three main polymorphs: calcite, aragonite and vaterite. Calcite is the most stable polymorph and usually exhibits a rhombohedral structure, while vaterite is mostly spherical. However, in the presence of bacteria (and maybe organics), this can be different (Qian et al., 2009). Therefore, it is difficult to judge the polymorph of the crystals only by their morphology. In current study, more efforts are put on overall filling of concrete cracks by bacterial precipitated CaCO_3_ to receive sealing and even healing of the cracks, which will contribute to a decrease of surface permeability. The morphology and polymorph of CaCO_3_ may have influence on their strength. This can be an interesting research aspect in later research. From the research of Bentz et al. (2015), it was found calcite had a higher bonding strength with cement hydrates than that of aragonite.

In summary, *B. sphaericus* spores were still viable after being encapsulated into the hydrogels in spite of the UV-irradiation and freeze drying and grinding. They can still germinate, decompose urea and precipitate CaCO_3_ on/in the hydrogel matrix. AM-H has a good bio-compatibility.

#### Cell-Entrapping Property of AM-H

Encapsulated spores can escape but most of them were entrapped in the matrix when the hydrogel was in water. The leakage results are shown in **Table 2**. The variations in the table indicate the standard errors of the results. It can be seen that around 0.39% of the spores leached out of the hydrogel after 3 days in water. Most of the leaching (more than 80%) occurred during the 2 min vigorous vortex, while only small amount of spores released in the still water after 1 and 3 days. No cells were detected in the solutions with AM-H.

**Table 2:** Results of the leakage test on AM-H encapsulated spores.

Type	Initial amount of spores (cells/mL)	Leached spores (cells/mL) and leaching ratio after first vortex		Leached spores (cells/mL) and leaching ratio after 1 days		Leached spores (cells/mL) and leaching ratio after 3 days	
AM-H	0	0	–	0	–	0	–
AM-HS	3.3^*^10^7^	(1.1 ± 0.1)^*^10^5^	0.33%	(1.2 ± 0.1)^*^10^5^	0.36%	(1.3 ± 0.1)^*^10^5^	0.39%

### Water Absorption Capacity

#### Swelling and Re-swelling Property of AM-H

**FIGURE 6:**
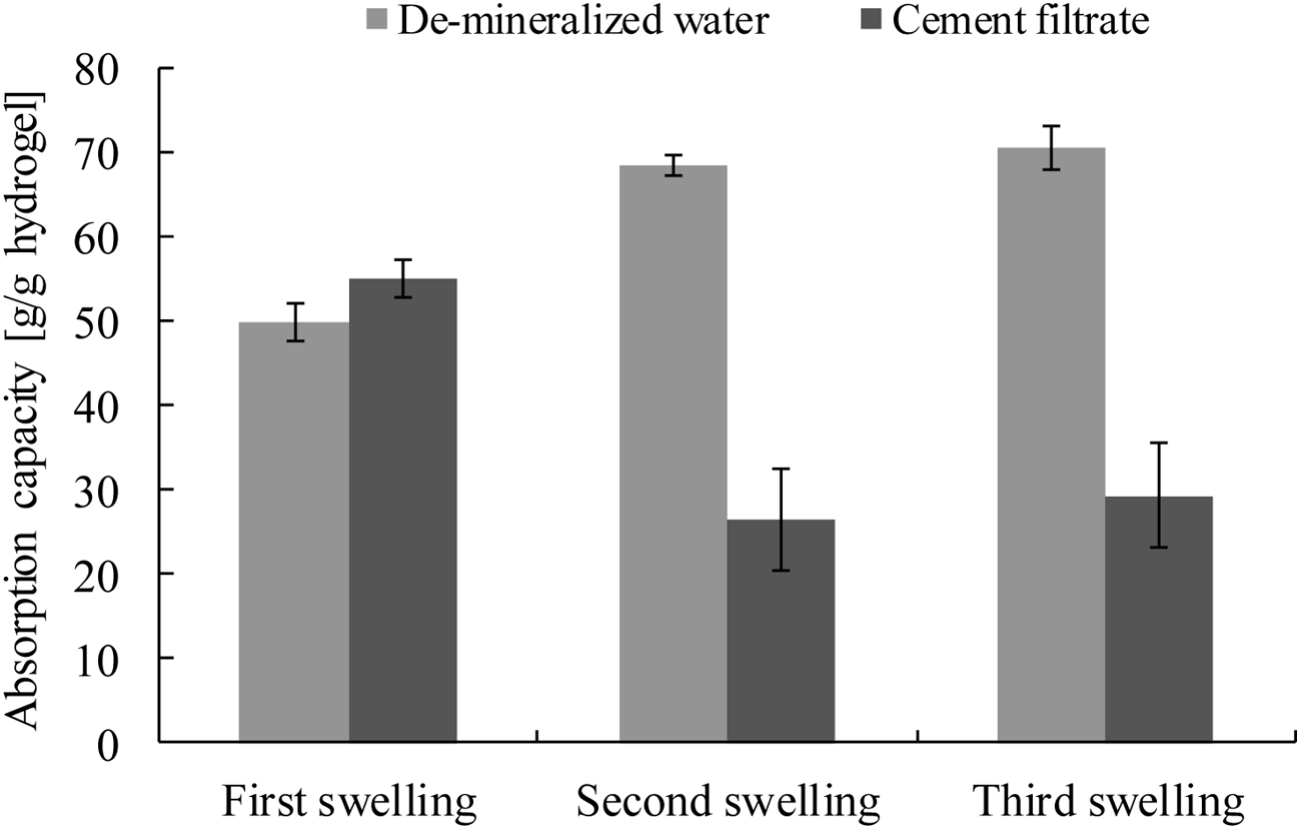
**Swelling properties of AM-H in de-mineralized water and in cement filtrate**.

The results of the swelling properties of modified alginate hydrogel in de-mineralized water and in cement filtrate are shown in **Figure 6** (with mean value and the standard deviation of the replicates). In de-mineralized water, the absorption capacity was 49.9 ± 2.3 g/g AM-H in the first test and increased to 68.6 ± 1.3 and 70.7 ± 2.5 g/g AM-H during the second and third swelling tests. In cement filtrate, the swelling capacity was similar (55.1 ± 2.2 g/g AM-H) to that in the de-mineralized water in the first test, but greatly decreased to 26.6 ± 5.6 and 29.4 ± 6.3 g/g AM-H during the second and third swelling tests. It can be seen that the swelling capacity of AM-H became stable after the second test. AM-H had a higher (around double) water absorption in de-mineralized water than in cement filtrate.

Due to a possible residue of the solvent in the material, the swelling properties are different in between the first and second swelling. Also, as the material was first freeze-dried (upon receiving) and the materials were vacuum dried afterward, this can also provide a small difference in values found. The value in de-mineralized water increases with swelling and reaches its stable end value. The value in cement filtrate, however, decreases. This is possibly due to the calcium binding in the polymeric structure of the modified alginate, which causes increased cross-linking. Afterward, the swelling property of the polymer is stable again. The remaining swelling property should be sufficient to promote bacterial self-healing when this hydrogel is used as an encapsulation material. The lower value found in cement filtrate is due to the charge screening effect of the negatively charged groups and the strong complexation in the hydrogel polymeric structure (Hancock and Martell, 1989; Snoeck et al., 2014b). Additionally, the Ca/Mg ions in cement filtrate can act as additional physical cross-linker because negative charged groups form around them, as mentioned before.

#### Moisture Uptake Capacity of AM-H

**FIGURE 7:**
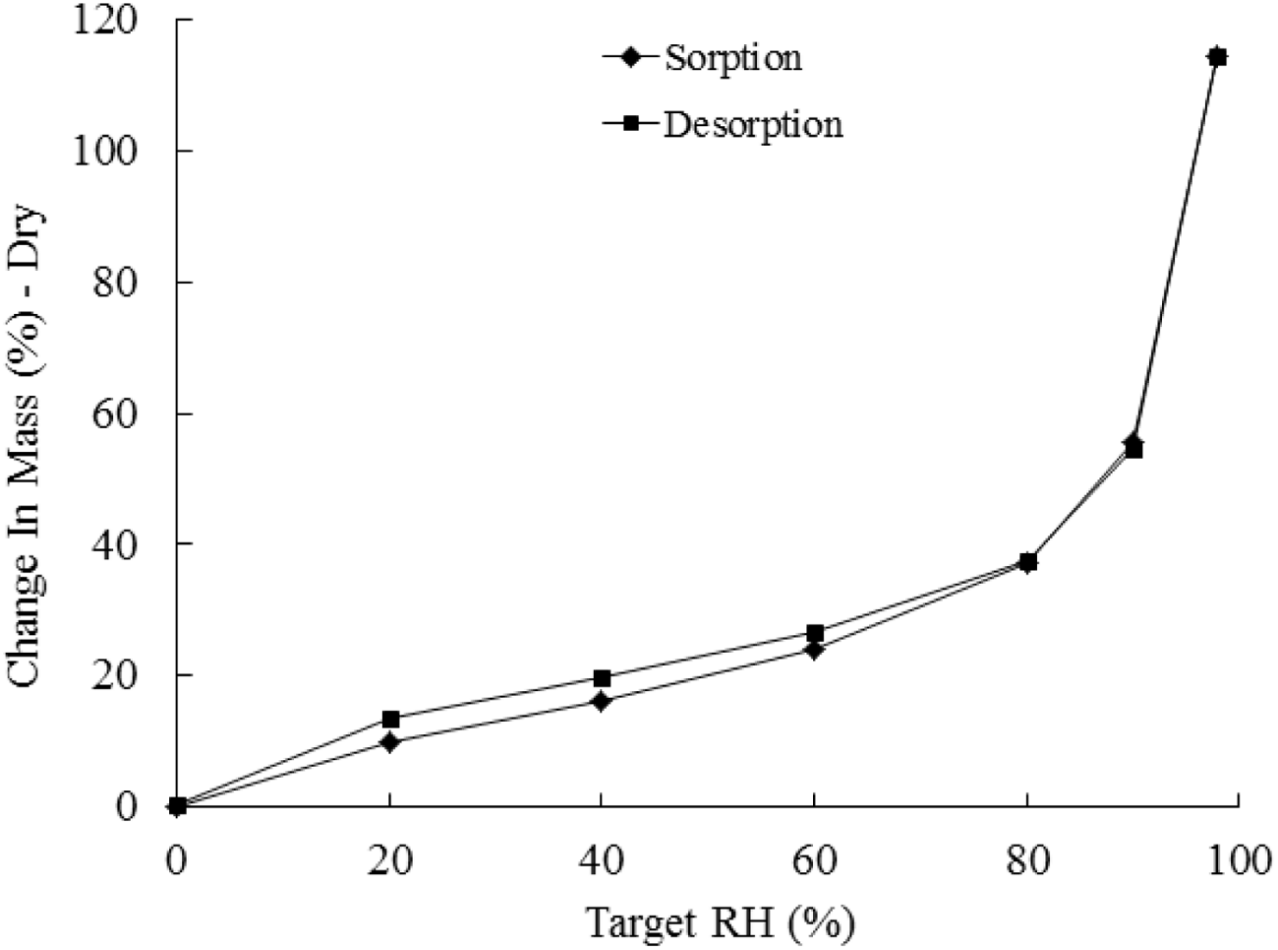
**Moisture absorption and desorption of AM-H under different relative humidities**.

To test the moisture uptake capacity of the AM-H, a DVS measurement was performed by measuring weight changes at controlled RHs. As shown in **Figure 7**, the AM-H can take up 27%, 54% and even up to 114% of their own weight at 60, 90, and 98% RH, respectively. Interestingly, there is a negligible level of hysteresis (up to 4%) during the desorption process. All moisture absorbed will thus again be able to desorb at equal RH levels. The absorbed moisture is useful for the bacterial activity.

### Influence of AM-H on the Mechanical Properties of Mortar Specimens

The workability of the fresh mortar mixture slightly decreased as the flow was lower after the addition of the AM-H. Reference mixtures show an average flow of 200 mm. When adding 0.5 m% of AM-H, this amount was 190 mm. When further increasing toward 1 m%, the flow reduced to 185 mm. It can be seen that the decrease in the workability is not noteworthy.

**FIGURE 8:**
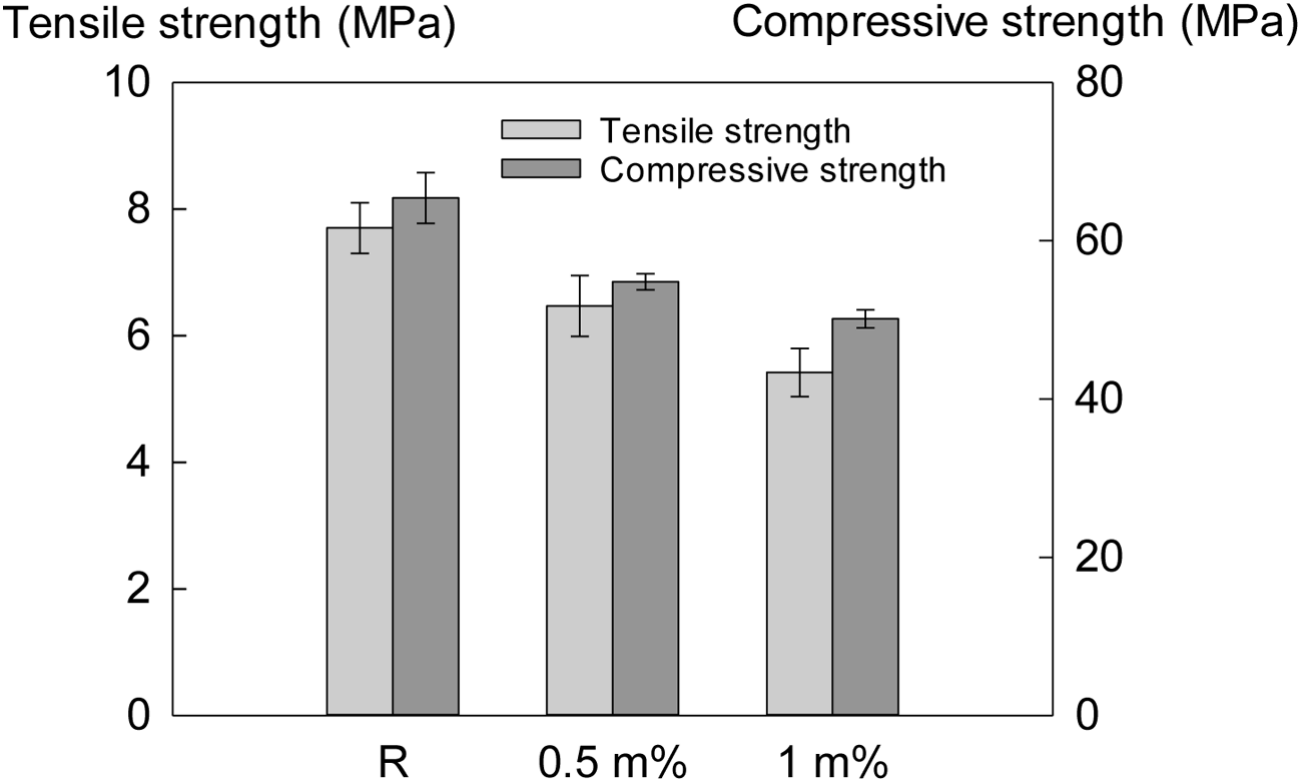
**Mechanical properties of the mortar specimens without and with different dosages of AM-H at the age of 28 days (The chart shows the mean value and standard deviation)**.

The addition of AM-H had a negative effect on the mechanical properties of the specimens. As shown in **Figure 8**, the strength of the specimens with the addition of 0.5 and 1% of the AM-H was decreased compared to the reference specimens: the tensile strength was decreased by 15.6 and 30%; the compressive strength was decreased by 16.2 and 23.4%, respectively. It can be seen that more strength was decreased at a higher dosage of addition (here is 1%), especially for the tensile strength.

### *In Situ* Bacterial Activity in Mortar Specimens

**FIGURE 9:**
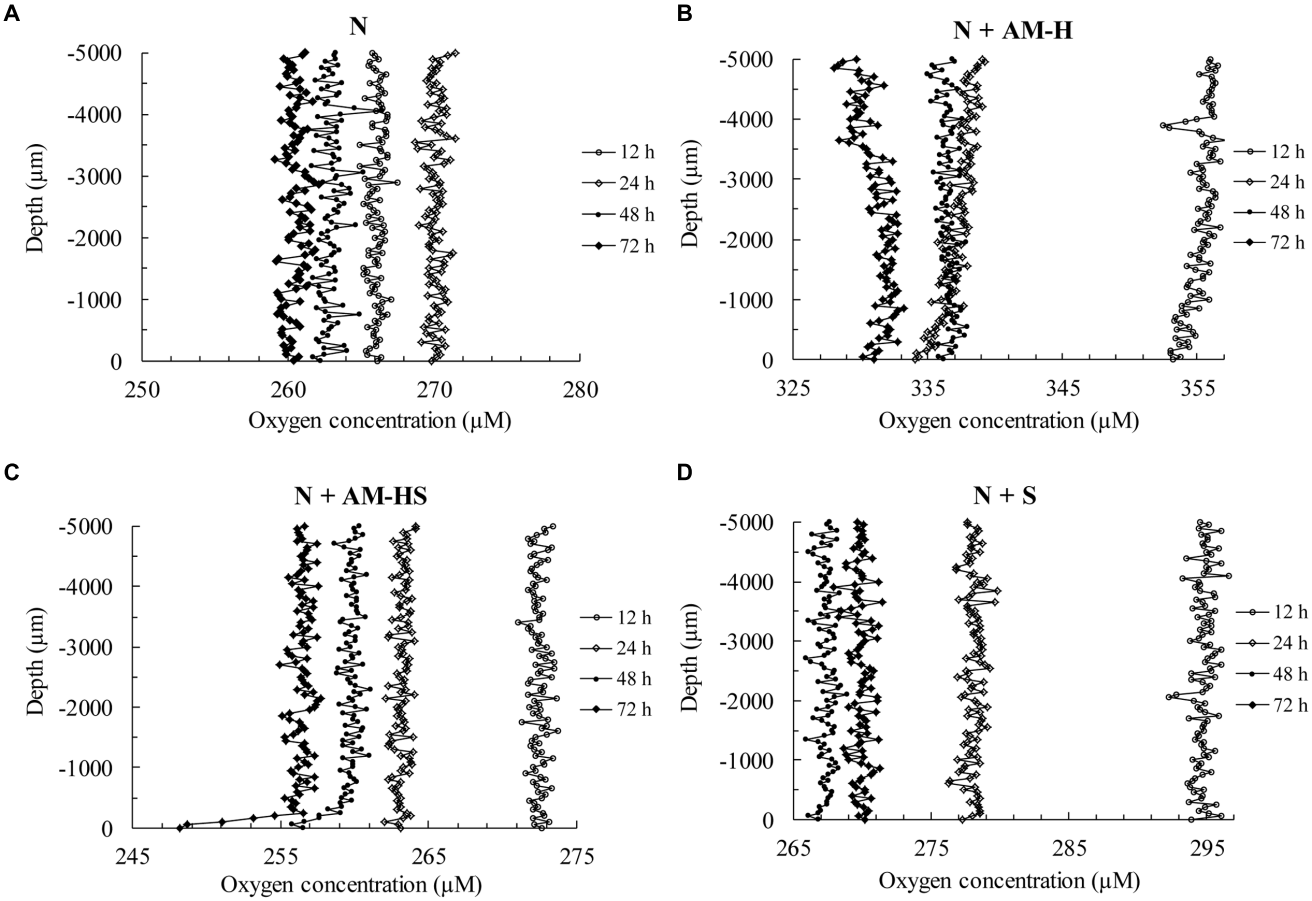
**Oxygen concentration profiles (at certain time intervals) toward the surfaces of different submerged mortar prisms [(A) specimen N; (B) specimen N+AM-H; (C) specimen N+AM-HS; (D) specimen N+S]**.

The oxygen concentration profiles were measured every 15 min for 3 days. It is redundant to show all the profiles. Therefore, only the ones at 12, 24, 48, and 72 h are shown in **Figure 9**. Each dot shows the real-time oxygen concentration at each layer. The measurements were done step by step and vertically from 5 mm above the surface (depth -5000 μm) till the surface (depth 0 μm). Generally, oxygen distrutes homogenously within a layer of 5 mm deep. If there is no oxygen consumption from bacterial activity (on the surface), the oxygen concentration along the whole depth will not have too much difference. In case of bacterial activity, a decrease of oxygen concentration will occur on the surface and in the layer which is very close to the surface.

For the specimen N (**Figure 9A**), the oxygen profiles at 12, 24, 48, and 72 h, shows quite homogenous and constant oxygen concentration along the whole depth, which indicates that no bacterial activity occurred at those moments. Similar results can be seen in the specimens N + AM-H (**Figure 9B**) and N + S (**Figure 9D**). In contrast, an obvious decrease of oxygen concentration starting from the depth of -500 μm can be seen on the profile of the specimen with encapsulated spores (N +AM-HS) at 48 and 72 h (**Figure 9C**), about 4 and 8 μM, respectively. This decrease was due to the bacterial activity.

**Figure 9** shows four oxygen profiles at certain time intervals, which only indicates the oxygen consumption at that specific moment. In order to have an overview of the development (from the beginning to the end) of the bacterial activity during the whole testing period, the amount of oxygen consumed in the boundary layer (from depth of -500 to 0 μm) was calculated (Δ[O_2_] = [O_2_]_-500__μm_ – [O_2_]_0__μm_) for each profile. The reason why taking the start of the at the depth of -500 μm is that the obvious oxygen concentration decrease mostly started from the depth of -500 μm for all the profiles of the specimen N +AM-HS (not shown here).

**FIGURE 10:**
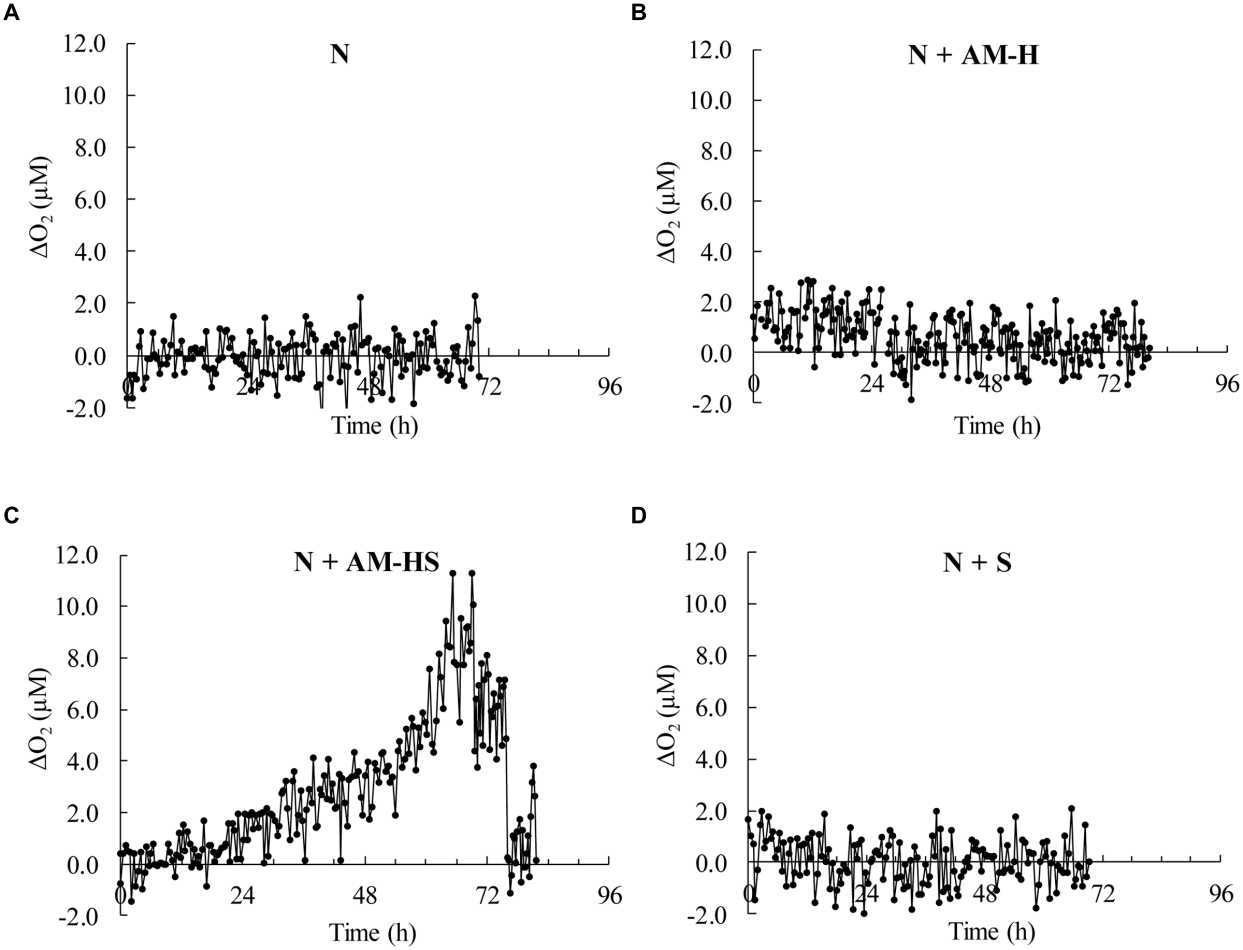
**Oxygen consumption in the boundary layer [the 0.5 mm water layer above the investigated location of the different prism surfaces, **(A)** specimen N; **(B)** specimen N+AM-H; **(C)** specimen N+AM-HS; **(D)** specimen N+S] as time went on**.

The oxygen consumption in the boundary layer of different specimens in function of time is shown in **Figure 10**. It can be seen that the specimens N, N + AM-H and N + S, had a very limited amount of oxygen consumption in the boundary layer, mainly in the range of -2∼2 μM in the whole testing period. Actually, this decrease was not due to oxygen consumption, but due to the variation of the oxygen concentration in the layer. That is why there were some negative values in the graphs. The specimen with encapsulated spores added shows a completely different graph. As shown in **Figure 10C**, the oxygen decrease was quite stable (varied a bit) in the first 24 h, and then gradually increased as time went on. Large consumption occurred from about the 60th h to the 72nd h, in the range of 6∼11 μM. After 72 h, the consumption started to decrease and tended to be stable at the end (-1∼2 μM). The considerable amount of oxygen consumption during 24–72 h was due to the bacterial activity on the damaged surface. It can be seen that encapsulation of the bacterial spores is thus very necessary to retain bacterial activity in mortar specimens.

## Discussion

### Viability of Modified Alginate Hydrogel Encapsulated *B. sphaericus* Spores

Bacterial viability normally can be investigated by use of fluorescence microscopy. Through a staining procedure, living and dead cells will show different colors under microscopy. This is easy for living cells but not the case for spores. It is difficult to stain spores and to differentiate the live/dead under microscopy. Furthermore, for the bacteria which are encapsulated, for example in hydrogels in our research, there could also be a limitation (interference of the hydrogel with the staining procedure) when applying this type of sample under microscopy. Therefore, we used an indirect way to determine the viability of the spores after encapsulated into the hydrogels. Urease activity and calcium carbonate precipitation are two most important properties for self-healing application. If the bacteria have urease activity and can precipitate calcium carbonate, they are definitely viable. The results from urease activity and calcium carbonate precipitation tests provided us reliable and meaningful data.

The importance of encapsulation of bacteria before addition into cement-based materials has been addressed and demonstrated in our previous research (Wang et al., 2012b, 2014a,c) and also by other researchers (Jonkers and Schlangen, 2009; Jonkers et al., 2010). One criterion for a suitable bacterial carrier is its biocompatibility, that is, bacteria should still retain their viability after encapsulation. In this study, the bacterial viability mainly refers to the urease activity and carbonate productivity of *B. sphaericus*. It can be seen from eq. (1) to eq. (2) that the prerequisite of the formation of bio-CaCO_3_ is bacterial urease activity, because urease catalyzed urea decomposition will produce carbonate ions, which are the necessary component for CaCO_3_ precipitation. Regarding the results of the viability test, it was found that *B. sphaericus spores* were still viable after being encapsulated into the modified alginate hydrogel. They can germinate and decompose urea (urease activity). AM-HS can precipitate considerable amount of CaCO_3_ on/in the hydrogel matrix (around 70% of the whole composites by weight). This biogenic mineral is the right repair material for concrete crack self-healing.

(1) $$CO{\left(NH_{2}\right)}_{2}+H_{2}O\overset{Bacterial\;\;urease\;}{⟶}CO_{3}^{2-}+NH_{4}^{+}$$

(2) $$Ca^{2+}+Co_{3}^{2-}\to CaCO_{3}$$

Another issue is the cell-entrapping property of the carrier. Encapsulated spores will be added into concrete during the mixing process. It is important to make sure that the spores or most of them will not be released from the hydrogel into the fresh mixture during the process of mixing and casting. A 2 min vigorous vortex was therefore applied first during the leakage test to mimic a real mixing process. Results show that most of the leakage (more than 80%) occurred during the vortex while only limited leakage happened in static water. The leakage is due to the network structure of the hydrogel matrix, which is sufficient to entrap cells in a static condition but maybe not strong enough to withstand all the shearing force during mixing. Nevertheless, most of the spores (99%) can be kept entrapped in the matrix. This indicates that some AM-H encapsulated spores would be released from the hydrogel during the concrete mixing process but most of them will stay inside the hydrogels and can be used for self-healing purposes. AM-H has a good cell-entrapping property for bacteria.

In summary, modified alginate hydrogel has a good biocompatibility with *B. sphaericus*.

### Compatibility of the AM-H with Cement-Based Matrix

The addition of AM-H resulted in a slight decrease in the workability of the fresh mixture. This is due to the partial uptake of mixing water by the hydrogel. However, the water absorption capacity of AM-H is much lower than some commercially available hydrogels which are called superabsorbent polymers (300 g water/g superabsorbent polymer). This is the reason why not much mixing water was absorbed and the influence on the workability is quite limited, which is acceptable from a practical point of view.

However, a more pronounced negative effect on the mechanical properties of the mortar occurred after adding the AM-H. This is mostly due to macropore formation by the hydrogels (Jensen and Hansen, 2002; Lura et al., 2006; Mechtcherine et al., 2009; Hasholt et al., 2012; Snoeck et al., 2014b). As they take up mixing water and only release it during hardening, an empty macropore remains. This pore negatively influences the strength. The bending strength is dependent on the total amount of air voids and macro pores in the cross-sectional area of the tensile plane. Due to the higher amount of macro pores in AM-H mixtures compared to the reference, the bending strength is lower. The released water can contribute to internal curing, inducing further hydration and densification (Hasholt et al., 2012). Even though there is a strength increase due to this internal curing, the localized strength reduction by the macro-pores is highlighted in the results. Increasing the amount of AM-H, also further decreases the strength. The compressive strength is also affected. As the macro pores are irregular in shape, the compressive loads are not transferred by dome action. This causes the compressive strength to be lower. The reduction in strength is higher when larger amounts of AM-H are used. However, it is worth to mention that the extra formation of the macropores caused by hydrogels can also be beneficial for increasing the freeze/thaw resistance of concrete (Mönnig and Lura, 2007). In practice, air entraining agent is added into concrete specially to induce some macropores to increase the freeze/thaw resistance. Generally, an air content of 5–7% (by volume) is recommended to obtain a good freeze/thaw resistance (NBN B15-001, 2004; Aci 201.2R, 2008). At this dosage, the strength of the concrete will decrease around 15–25% (Pinto and Hover, 2001). The negative effect on the strength is mostly counteracted by the positive effect on the freeze/thaw resistance. Furthermore, the reduction in strength caused by the addition of AM-H is acceptable comparing to the strength decrease from incorporating other types of bacterial carriers. In our previous study, expanded clay (kind of porous aggregates) microcapsules and pluronic-hydrogel were used to encapsulate bacteria for self-healing purpose (Ameye, 2012; Wang, 2013; Wang et al., 2014a). The strength of the mortar specimens was decreased by 20–30% when using expanded clay and microcapsules, and around 50% for the ones with pluronic-hydrogel. Regarding the problem of strength reduction caused by the addition of the self-healing agents, a solution can be that extra functional agents will be added to increase the mechanical strength of the concrete, which is often the case in practice.

### *In Situ* Bacterial Activity and Potential Application for Realistic Self-Healing Concrete

Bacterial-based self-healing relies on the calcium carbonate precipitation induced by the bacteria in crack zone. In the previous study, enhanced healing efficiency has been obtained regarding crack filling and water penetration resistance for specimens containing encapsulated bacteria, which provides indirect proof of the superiority of using carbonate precipitating bacteria for crack self-repair. However, calcium carbonate also can form from concrete carbonation (see Equation 3), which may also contribute to crack filling. It is hard to differentiate the biogenic-CaCO_3_ from abiotic-CaCO_3_. Therefore, *in situ* bacterial activity in crack zone can be a direct demonstration for the bacterial-contributed self-healing.

(3) $$Ca{\left(OH\right)}_{2}+CO_{2}\to CaCO_{3}+H_{2}O$$

Cracks may appear at any period during the service time, healing agents should have a long lifespan for the crack repair at any time. *B. sphaericus* spores instead of vegetative cells were therefore used in the study because spores have much longer survival time and much higher resistance in a harsh condition (concrete environment) than vegetative cells (Setlow, 2000). However, spores are the dormant state of living cells. Upon cracking, the embedded *B. sphaericus* spores have to be activated first. One aim of this study was to monitor the *in situ* bacterial activity on the damaged mortar surface. Oxygen is consumed during the germination of *B. sphaericus* spores. Therefore, whether the spores (encapsulated or not encapsulated) in/on the damaged surface become active or not can be indicated by the oxygen consumption on the surface. It was found that only the specimens with AM-HS added showed considerable oxygen consumption, indicating the bacterial activity on the damaged surface. This implies that the encapsulated spores are still viable and can become active after damage occurs. The germination of spores started not immediately at the beginning but after 24 h, and oxygen consumption rate varied in the whole period. Although the oxygen consumption almost stopped after 3 days (**Figure 10C**), this does not mean that the bacterial activity also stopped. After germination, the active cells may decompose urea if there is urea available in the surroundings and CaCO_3_ precipitation will then form with the presence of Ca^2+^. The urea decomposition by bacteria is more an enzymatic reaction (see Equation 1), which does not consume oxygen.

In this study, specimens with non-encapsulated spores added did not show any bacterial activity on the damaged surface. It is assumed that the free spores had lost their viability (cannot germinate) after being incorporated into the specimens. Compared to the specimens with encapsulated spores, it can be concluded that encapsulation of spores is of crucial importance to retain bacterial viability in concrete. Regarding AM-H, even though with some leakage, most of the spores can be protected in the hydrogel matrix and revive their activity in the damaged zone when exposed to oxygen and water. Water is another necessary element for spores germination and later bacterial precipitation. Continuous and sufficient water supply is a key factor for a good healing efficiency. During the test period, the specimens were fully immersed in a water bath to facilitate the revival of bacterial activity. However, in many practical occasions, full immersion of specimens or structures is not possible. The AM-H may help in a realistic situation. Swelling and DVS test results show that AM-H has a good water absorption and moisture uptake capacity. This implies that even in the constructions in normal humid environments, AM-H can be used as a moisture reservoir to retain high amount of moisture from the air (more than its own weight), depending on the RH of the surroundings. The absorbed moisture can be supplied for bacterial activity to precipitate CaCO_3_, and diffuses to the remaining unreacted cement particles which contribute to the autogenous healing of concrete. This illustrates the great potential of using carbonate precipitating bacteria in combination with AM-H for realistic self-healing concrete. Further research will be focused on the investigation of self-healing efficiency by use of AM-HS in mortar and concrete.

## Conclusion

Carbonate precipitating bacterium *B. sphaericus* was successfully encapsulated into a modified alginate based hydrogel, which was proven to have a good biocompatibility with the bacteria. The viability of the bacterial spores was retained after encapsulation regarding the ureolytic activity and the calcium carbonate precipitation on/in the hydrogel matrix. The AM-H also has a good cell-entrapping capacity, which can keep more than 90% of the encapsulated spores from releasing during the mixing process. Besides, the AM-H has a good water absorption and moisture uptake capacity. All these are beneficial for the bacteria-based self-healing in concrete although the addition of the hydrogel can cause some (acceptable) strength reduction (16∼23%). The limited strength reduction of the virgin samples, however, can be compensated if needed (by adding more cement, better particle packing, less water, adding functional agents, etc.). Meanwhile, the compatibility between hydrogels and cement based matrix can also be further improved in future research to have less negative effect on the strength. In addition, depending of the application, the (small) strength reduction might not be a problem, e.g., for use in irrigation canals.

For the first time, *in situ B. sphaericus* activity on the damage mortar surface was demonstrated by an oxygen consumption test. The activity was only found in the specimens with encapsulated spores; while no activity was detected in the ones with non-encapsulated spores. This implies that AM-H has a protective effect for bacteria. To conclude, AM-H encapsulated bacteria has a great potential to be applied for self-healing concrete.

## Author Contributions

JW was involved in work conception, experimental design, experimental work, data analysis, manuscript writing and revision, and final manuscript approval. AM was involved in experimental work, data analysis, manuscript writing and revision, and final manuscript approval. DS was involved in experimental work, data analysis, manuscript writing and revision, and final manuscript approval. VW was involved in experimental work, data analysis, critical manuscript revision, and final manuscript approval. SVV provided scientific expertise and guidance on hydrogel chemistry, critical manuscript revision, and final manuscript approval. NB provided scientific expertise and guidance on microbiology, critical manuscript revision, and final manuscript approval. NDB was involved in work conception, discussion on the data interpretation, critical manuscript revision, and final manuscript approval. All authors agree for work accountability.

## Conflict of Interest Statement

The authors declare that the research was conducted in the absence of any commercial or financial relationships that could be construed as a potential conflict of interest.
